# A case report of adult-onset Alexander disease clinically presenting as Parkinson’s disease: is the comorbidity associated with genetic susceptibility?

**DOI:** 10.1186/s12883-020-1616-8

**Published:** 2020-01-17

**Authors:** Jongkyu Park, Sung-Tae Park, Jieun Kim, Kyum-Yil Kwon

**Affiliations:** 1 Department of Neurology, Soonchunhyang University Cheonan Hospital, Soonchunhyang University College of Medicine, Cheonan, Republic of Korea; 2 Department of Radiology, Soonchunhyang University Seoul Hospital, Soonchunhyang University College of Medicine, Seoul, Republic of Korea; 3 Department of Laboratory Medicine, Soonchunhyang University Seoul Hospital, Soonchunhyang University College of Medicine, Seoul, Republic of Korea; 4 Department of Neurology, Soonchunhyang University Seoul Hospital, Soonchunhyang University College of Medicine, 59 Daesagwan-ro, Yongsan-gu, Seoul, 04401 Republic of Korea

**Keywords:** Alexander disease, Parkinson’s disease, MRI, FP-CIT PET

## Abstract

**Background:**

Alexander disease is a rare neurological disease characterized by progressive spastic quadriparesis and bulbar palsy. Moreover, certain patients with adult-onset Alexander disease were often misdiagnosed as other neurodegenerative disorders.

**Case presentation:**

Herein, we report an adult a 58-year-old woman presented with typical parkinsonism with good levodopa-responsiveness. The patient’s dopamine transporter scanning showed significant striatal depletion, while her brain magnetic resonance imaging revealed bilateral tadpole shape of medulla oblongata and bilateral high signal intensity at both cerebellar dentate nuclei in T2-weighted images, suggesting the possibility of a genetic disorder beyond Parkinson’s disease. The patient’s genetic test resulted in known pathogenic glial fibrillary acidic protein variant, indicating Alexander disease.

**Conclusion:**

This unique case highlights genetically diagnosed Alexander disease may present with clinical Parkinson’s disease.

## Background

Alexander disease is a neurological disease that causes leukodystrophy and neuronal loss of brain, due to mutation of glial fibrillary acidic protein (GFAP) gene. When age of onset is high, bulbar symptoms and cerebellar dysfunctions develop gradually, requiring discrimination from adult-onset neurodegenerative disorders [1]. Herein, we report a unique case of genetically diagnosed Alexander disease comorbid, with clinically diagnosed Parkinson’s disease (PD).

## Case presentation

We describe a 58-year-old Korean woman who developed tremor 4 months ago. Family history was negative. The patient was taking levothyroxine 0.175 mg daily, after thyroidectomy for 1 year. On neurological examination, she showed mild rigidity and bradykinesia, more predominant in left limbs, compared to right limbs. Rest tremor was observed only in left limbs and was more predominant in the leg. Neither postural instability, nor ataxia was checked, while brisk reflexes were present on both lower limbs without pathologic reflexes. The patient had no problem with social activity, although detailed neuropsychological assessment resulted in mild cognitive impairment.

**Fig. 1:**
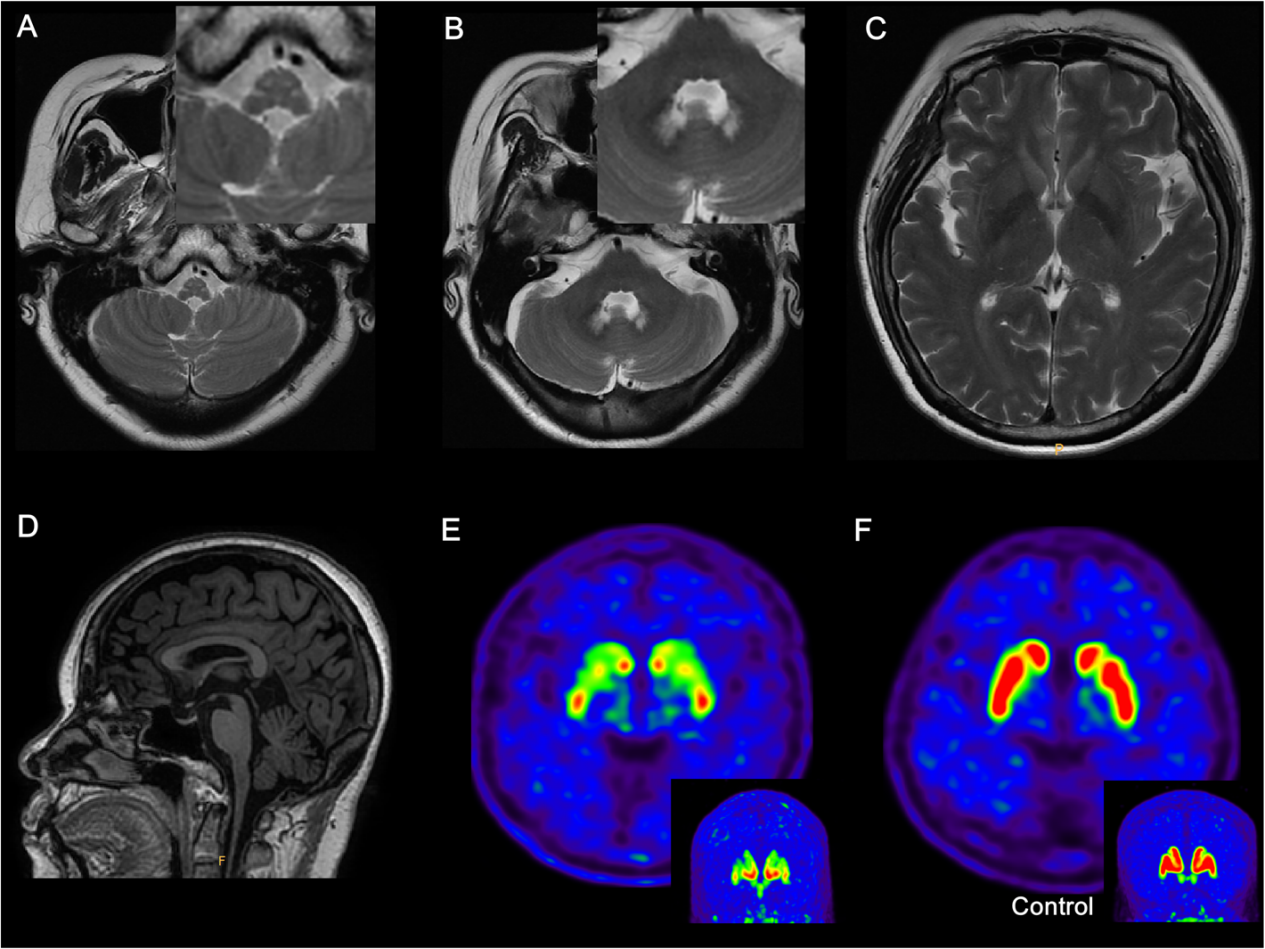
Brain MRI and FP-CIT PET imaging of the patient. **a**, **b** Brain MRI with T2-weighted axial images show typical bilateral high signal intensity at both medulla oblongata and cerebellar dentate nuclei. **c** No major lesions in the basal ganglia and periventricular white matter. **d** A T1-weighted sagittal image shows tadpole shape cervicomedullary atrophy. **e** 3-dimensional [18F] FP-CIT PET shows severely decreased dopaminergic uptake in bilateral putamen and caudate nucleus with a rostrocaudal gradient. **f** FP-CIT PET in control shows normal dopaminergic uptake in bilateral putamen and caudate nucleus

Routine laboratory work ups were unremarkable. Brain magnetic resonance imaging (MRI) with T2-weighted images show bilaterally mottled high signal intensities at medulla oblongata and bilateral high signal intensity at both cerebellar dentate nucleus (Fig. 1a and b). Meanwhile, there is no abnormal change in basal ganglia (Fig. 1c). In addition, a sagittal T1-weighted image demonstrates considerable cervicomedullary atrophy with intact pons (tadpole sign, Fig. 1d). 18F- 2b-carbomethoxy-3b-(4-iodophenyl)-N-(3-fluoropropyl) nortropane (FP-CIT) positron emission tomography (PET) of the patient show severely decreased FP-CIT binding not only in the bilateral putamen, but also in the bilateral caudate nucleus with a rostrocaudal gradient (Fig. 1e), compared with normal FP-CIT imaging finding of control (Fig. 1f). Anti-parkinsonian medications improved her motor symptoms considerably. However, based on abnormalities from the brain MRI, we could not exclude the possibility of comorbidity. We further performed next generation sequencing of customized panel, targeting 95 genes associated neurologic disorders by target capture method, resulting in known pathogenic heterozygous p.Arg70Trp variant (NM_002055.4:c.208C > T) in GFAP gene [2], confirmed by Sanger sequencing method. Until now, the patient has been followed up for 18 months, and she has shown a good responsiveness to anti-parkinsonian medications.

## Discussion and conclusions

We report an adult patient with levodopa-responsive parkinsonism and known pathogenic GFAP variant. This patient did not have typical symptoms suggestive of Alexander disease. The adult-onset Alexander disease has various clinical features, mainly speech abnormalities, swallowing difficulties, frequent vomiting, lower limb spasticity and ataxia [3]. It can be found incidentally at autopsy or molecularly confirmed cases without a prior history and it is possible that the patient may never develop symptoms related to this genetic diagnosis [4]. It may be reasonable to infer that abnormalities of the brain MRI are mild, and it is difficult for them to develop symptoms, or they may develop for a lengthy time, and become chronic adaptation or compensation. In contrast, the patient showed not only typical parkinsonism, but also good levodopa responsiveness, indicating a clinical diagnosis of PD. Moreover, this was highly supported by FP-CIT PET findings of presynaptic dopaminergic depletion. To the best of our knowledge, a case of Alexander disease, with both typical parkinsonism and striatal dopamine depletion, has not been reported.

The GFAP variant, when expressed in older ages, causes neuronal loss in the brain especially in the brain stem [5]. In some reports, neuronal loss in basal ganglia are found in adult-onset GFAP variant [6–8], that presence of striatonigral neuronal loss on FP-CIT PET with GFAP variant was first described in this case. An unusual pattern of dopamine depletion with a rostrocaudal gradient in the putamen and caudate nucleus were seen, unlike in patients with PD which shows preferential depletion in posterior putamen with relative sparing of caudate nucleus [9]. The atypical dopamine depletion pattern has not been described or investigated in other Alexander disease patients in the literature and that it could also relate to the interaction of co-pathology.

We present an unusual case wherein clinical PD, and genetic Alexander disease coexist. If MRI findings suggestive of leukoencephalopathy-related disorders are observed in patients with PD, clinicians need to further investigate the possibility of genetic variant of adult-onset leukoencephalopathy, including Alexander disease.

## Data Availability

The dataset supporting the conclusions of this article is included within the article.

## Abbreviations

FP-CIT: 2b-carbomethoxy-3b-(4-iodophenyl)-N-(3-fluoropropyl) nortropane
GFAP: Glial fibrillary acidic protein
MRI: Magnetic resonance imaging
PD: Parkinson’s disease
PET: Positron emission tomography (PET)

## References

[1] Ahmad O, Rowe DB (2015). Adult-onset Alexander’s disease mimicking degenerative disease. Pract Neurol.

[2] Yoshida T, Sasayama H, Mizuta I, Okamoto Y, Yoshida M, Riku Y, Hayashi Y, Yonezu T, Takata Y, Ohnari K, Okuda S, Aiba I, Nakagawa M (2011). Glial fibrillary acidic protein mutations in adult-onset Alexander disease: clinical features observed in 12 Japanese patients. Acta Neurol Scand.

[3] Graff-Radford J, Schwartz S, Gavrilova RH, Lachance DH, Kumar N (2014). Neuroimaging and clinical features in type II (late-onset) Alexander disease. Neurology.

[4] Gorospe JR, Naidu S, Johnson AB, Puri V, Raymond GV, Jenkins SD, Pedersen RC, Lewis D, Knowles P, Fernandez R, De Vivo D, van der Knaap MS, Messing A, Brenner M, Hoffman EP (2002). Molecular findings in symptomatic and pre-symptomatic Alexander disease patients. Neurology.

[5] Sosunov A, Olabarria M, Goldman JE (2018). Alexander disease: an astrocytopathy that produces a leukodystrophy. Brain Pathol.

[6] Namekawa M, Takiyama Y, Honda J, Shimazaki H, Sakoe K, Nakano I (2010). Adult-onset Alexander disease with typical “tadpole” brainstem atrophy and unusual bilateral basal ganglia involvement: a case report and review of the literature. BMC Neurol.

[7] Schmidt H, Kretzschmar B, Lingor P, Pauli S, Scheramm P, Otto M, Ohlenbusch A, Brockmann K (2013). Acute on set of adult Alexander disease. J Neurol Sci.

[8] Elmali AD, Cetincelik U, Islak C, Uzun AN, Karaali SF, Yalcinkaya C (2016). Familial adult-onset Alexander disease: clinical and neuroradiological findings of three cases. Noro Psikiyatry Ars.

[9] Oh M, Kim JS, Kim JY, Shin KH, Park SH, Kim HO, Moon DH, Oh SJ, Chung SJ, Lee CS (2012). Subregional patterns of preferential striatal dopamine transporter loss differ in Parkinson disease, progressive supranuclear palsy, and multiple-system atrophy. J Nucl Med.

